# Placental Amniotic Epithelial Cells and Their Therapeutic Potential in Liver Diseases

**DOI:** 10.3389/fmed.2014.00048

**Published:** 2014-12-08

**Authors:** Asli Ceren Tahan, Veysel Tahan

**Affiliations:** ^1^University of Iowa College of Science, Iowa City, IA, USA; ^2^Department of Gastroenterology, University of Iowa, Iowa City, IA, USA

**Keywords:** placental amniotic epithelial cells, therapy, liver diseases, stem cells, placenta

## Abstract

As a unique source of stem cells, there is a growing interest in amniotic epithelial (AE) cells. Placenta is readily available; in fact, it is often discarded following delivery. As such, it is without the ethical concerns of embryonic stem cells. Further advantages to AE include that AE cells do not demonstrate tumorigenicity upon transplantation, and are gifted with immunomodulatory and anti-inflammatory properties. Thus, AE cells have exceptional features for use as cell-based therapies for liver disease.

## Introduction

Amniotic epithelial (AE) cell studies in therapy in liver disease in several ways: (A) AE cell therapy is an alternative or bridge to solid organ transplantation as shown in both animal models and clinical cases. (B) Transplanted cells can provide functional liver support while the recipient’s liver regenerates in acute liver failure patients and may provide a certain time as a bridge until orthotopic liver transplantation (OLT). (C) They have valuable effect in substituting a missing enzyme function in metabolic diseases with an aim of avoiding OLT. (D) Hepatocytes, embryonic stem cells, mesenchymal stromal cells, placental AE cells, and induced pluripotent stem cells are the most capable cell types that can be used in liver diseases. Thus, placental AE cell studies and its therapy show promise in liver diseases (1–3). We aimed to review current placental AE cell studies and its therapy in liver diseases.

## How and Why Can AE Cells be Used?

**Figure 1:**
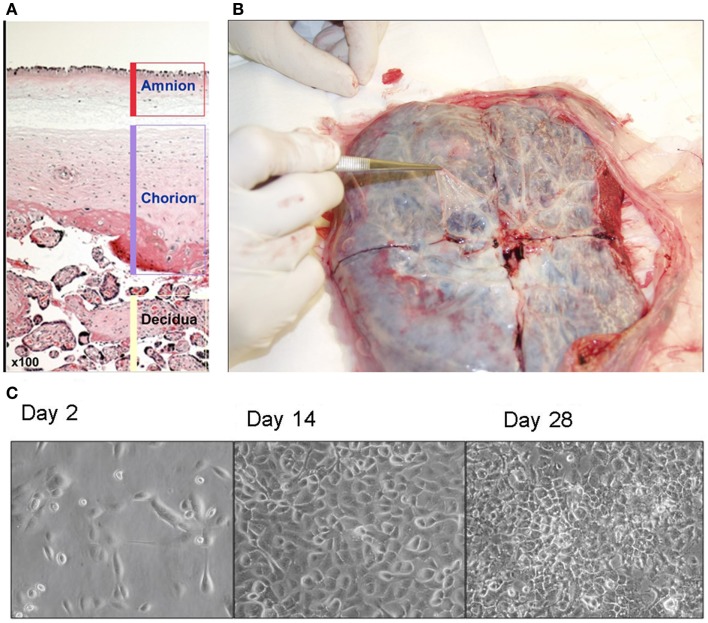
**(A)** Human placenta layers: amnion, chorion, and decidua. Amniotic layer is composed of a single-celled epithelial layer and a deeper mesodermal layer. Chorionic layer is composed of a mesodermal layer and a trophoblast layer. **(B)** Isolation of amnion membrane from placenta. The maternal side of placenta is placed face down and a shallow X-shaped incision is made through the center of the placenta. The thin, nearly transparent amnion membrane is then peeled starting at the center of the cut and progressing outward. **(C)** Morphology of amniotic epithelial cells in culture (×40).

Amniotic epithelial cells can be isolated from a full term placenta following live birth. This readily available tissue is normally discarded. Although any placenta is considered a useful source of AE cells, AE cell isolations are typically obtained from cesarean section deliveries due to sterility concerns. Perhaps at some future date placental stem cells could be isolated from all term births and cryopreserved in a cell bank for future use (3, 4). It is hard to imagine religious or political opposition for such a benign source of stem cells. A human placenta contains three different layers: amnion, chorion, and decidua (Figure 1A). The amniotic layer is composed of cuboidal and columnar cells and a deeper mesodermal layer composed of an upper compact acellular layer and a lower fibroblast-containing layer. The amnion layer is derived from pluripotent epiblast that gives rise to all three germ layers of embryo. Amnion is derived at a time when the epiblast remains pluripotent, and AE cells hold some of these characteristics. AE cells can be isolated from a full term placenta. Placenta is a readily available tissue and is generally discharged after delivery. It provides a valuable source of pluripotent stem cells that cells are plentiful and free from most ethical and religious concerns. Therefore, in practice, placental stem cells can be isolated from all term births and cryopreserved in a cell bank for future use (3, 4).

## Stem Cell Characteristics of AE Cells

Amniotic epithelial cells have stem cell molecular markers as OCT-4, Nanog, SOX-2, and Rex-1. AE cells have some advantage over embryonic stem cells; AE cells do not need feeder cell layers to preserve OCT-4 and Nanog expression in their cultures (4, 5). Since AE cells do not have telomerase reverse transcriptase, they show a stable karyotype and do not develop any tumors. Other stem cells have a common risk of teratoma development, when transplanted (2, 5). AE cells are derived from neonatal tissue and therefore have lesser DNA damage (6). Amnion does not express HLA class II antigens; therefore, AE cells can bypass the immune system. AE cells can inhibit inflammation and proliferation of T- and B-cells *in vitro* by their secretions (7). AE cell transplantation in some volunteers did not cause any immunological reactions. With their non-tumorogenic characteristics (8–10), AE cells have beneficial effects similar to embryonic stem cells but without their dangerous side effects.

## AE Cell Isolation and Differentiation Methods

Amnion membrane can be peeled away from the chorion (Figure 1B). It is the source of AE cells and amniotic mesenchymal (AM) fibroblasts. We recommend our study group’s isolation protocol (11). Briefly, amnion membrane is washed at least three times to eliminate blood cells. Then trypsin can be applied to release AE cells from AM fibroblasts and connective tissue. Then cell suspension can be centrifuged to pellet AE cells and resuspended in standard. Cell viability is calculated by trypan blue stain on hemocytometer. Our group’s proposed method can be used to isolate 80–300 million cells from one term placenta (11), which is a plentiful supply, indeed. Epidermal growth factor (EGF) is used in AE cell cultures to promote their growth and differentiation *in vitro* (Figure 1C) (4). Our study group also showed an efficient method to differentiate AE cells to hepatocyte-like cells (2), which expressed many hepatic marker genes. Since these hepatocyte-like cells express CYP3A4 and CYP3A7, we can hypothesize that AE cells are likely to differentiate along a pathway equivalent to human fetal liver. Furthermore, the changing ratio of CYP3A4 to CYP3A7 in their growing period suggests that cells are developing mature hepatocytes (12). Human AE cell transplantation to mice liver also exhibited human hepatic genes expressions such as albumin, cytochromes, and alpha-1 antitrypsin (2, 5).

## Why are AE Cells Preferred Over Other Sources of Stem Cells?

Because, AE cells have stem cell characteristics with low immunogenicity, and anti-inflammatory possessions, they show exciting potential in the regenerative medicine (Figure S1 in Supplementary Material). AE cells exhibit hepatic gene expression and functions close to mature hepatocytes level following their transplantation into the liver of severely combined immunodeficiency (SCID) mice, which prove their differentiation into hepatocyte-like cells once engrafted in the parenchyma (5, 11). Recent studies have shown that AE cells can engraft into the livers of immunocompromised mouse of liver and improve any damage by reducing hepatic fibrosis, inflammation, and apoptosis (2, 13). AE cell transplantation successfully corrected lysosomal storage diseases in clinics without any adverse effects (9, 10, 14). AE cell transplantation can rescue a mouse model of intermediate maple syrup urine disease (iMSUD) (1), which is a congenital disorder characterized by deficiency of the branched-chain keto-acid dehydrogenase (BCKDH) enzyme complex and elevated branched-chain amino acids (BCAA) (15). In our group’s another study, iMSUD model, mice were given multiple injections of AE cells directly into liver parenchyma. Normally, untreated iMSUD mice can grow sickly and all die prior to 27 days of age. IMSUD-treated mice could display improved BCKDH enzyme activity, reduced BCAA, and other relevant metabolites, they could gain weight as healthy wild type littermates, and more than 70% of AE transplanted iMSUD animals survived (1). Manuelpillai et al. transplanted human AE cells to mice via tail vein on carbon tetrachloride (CCL4) model. The AE treatment improved ALT levels, hepatic stellate cell activation, and hepatic fibrosis (13). Zhang et al. used intrasplenic route to transplant AE cells in CCL4-treated mouse. They also showed improvement on markers of ALT, apoptosis, and fibrosis (16). Hodge et al. recently have showed that human AE treatment suppresses markers of activation, proliferation, and fibrosis in human hepatic stellate cells in co-cultures. AE therapy also induces apoptosis of hepatic stellate cells (17). Niemann–Pick disease is a hereditary disorder of sphingomyelinase deficiency that leads to excessive intracellular lipid accumulation and causes severe liver damage and neuronal degeneration. Hong et al. used human AE cells as a source of enzyme replacement in a mouse model of Niemann–Pick disease. They transplanted half a million AE cells every other week from 5 weeks of age. The treatment dramatically prolonged the survival of the treatment group, and resulted in improvement of tissue damage (18). Bembi et al. reported five type B Niemann–Pick patients, in which AE cells supplied exogenous sphingomyelinase, following the transplantation. AE transplantation normalized patients’ urinal sphingomyelin and phospholipid levels (19).

## Benefits of AE Cells

Amniotic epithelial cells are relatively easy to isolate and do not require a complicated laboratory set up (4). Approximately 100 million cells per placenta can be isolated, and AE cells can grow fast. Their smaller size, compared to hepatocytes brings technical advantage for their injection and engraftment. AE cells can be cryopreserved long term. AE cells can have beneficial effects of targeted cells and can transform clinically relevant cells. They also secrete anti-inflammatory factors and are non-immunogenic. Most likely, current umbilical cord blood stem cell guidelines would be appropriate to use as a model for banking processes for AE cells (20). We do not know whether hepatic differentiation prior to transplantation is necessary for an effective method of treatment. Since their safety has been clearly established, AE cells with their potential effects continue to be a therapeutic hope in liver diseases (Table S1 in Supplementary Material).

## Why are AE Cells Preferred Over Other Sources of Stem Cells?

Currently, we need alternative cell sources to hepatocytes. Hepatocytes are very vulnerable cells and cannot survive mostly no more than couple of weeks on the cultures. They are difficult to isolate and their cryopreservation is not successful to keep their viability high. Their larger size, compared to stem cells can cause technical disadvantage for their injection and infusion. AE cells do not have the disadvantages of hepatocytes. AE cells do not have any side effects of other types of stem cells either. They are immunogenic and are not tumorogenic in both undifferentiated and differentiated stages. All other stem cell types have tumorigenicity risks associated with their use *in vivo*. They do not cause acute rejection. AE stem cells for cell transplantation are currently in preclinical or early clinical stages. They are obviously the harmless alternate to hepatocytes for therapies of liver diseases. AE type stem therapy is a new hope substitute for hepatocytes in liver diseases.

## In Conclusion

In conclusion, current studies’ results suggest that AE cells isolated from discarded placenta may be an abundant, non-controversial, and safe source of stem cells for regenerative medicine. AE cell transplantation can be an alternative therapy to OLT in the near future to avoid the increasing amount of patient deaths due to insufficient numbers of available livers and/or long organ wait times. Cell transplantation has shown a great deal of promise, and the progress made over the past several decades of preclinical and clinical studies provides a growing amount of rationale for its use to treat a variety of liver disorders. Currently, studies with appropriate Neimann–Pick disease (18), acute liver failure (21) and liver fibrosis (22, 23) models, and their correction by the transplantation of human AE stem cells are motivating the movement of banking of AE cells so that they can be used in the clinic for transplantation to treat liver diseases.

## Conflict of Interest Statement

The authors declare that the research was conducted in the absence of any commercial or financial relationships that could be construed as a potential conflict of interest.

## Supplementary Material

The Supplementary Material for this article can be found online at http://www.frontiersin.org/Journal/10.3389/fmed.2014.00048/abstract
