# Supplementary material for: Placental Amniotic Epithelial Cells and Their Therapeutic Potential in Liver Diseases
Source: Front Med (Lausanne). 2014 Dec 8;1:48. doi: 10.3389/fmed.2014.00048 (PMC4291892; doi:10.3389/fmed.2014.00048)
Supplement: Supplementary file 1 [file Table_1.PDF]

Table 1. Studies using amniotic epithelial cells in liver diseases.

Liver Disease	Outcome	References
Maple syrup urine disease mouse model	Human amniotic cell transplantation lengthened survival	Skvorak et al. Hepatology 2013 (1)
Niemann-Pick disease, a patient	Repeated implantations of human amniotic sheets provided successful enzyme replacement	Scaggiante et al. Transplantation 1987 (14)
Niemann-Pick disease type C1 mouse model	Extended the life span and improved the weight	Hong et al. Cytotherapy 2012 (18)
Niemann-Pick disease type B, 5 patients	Sphingomyelinase activity improved to heterozygous level values	Bembi et al. Am J Med Genet 1992 (19)
Liver fibrosis in immunocompetent CCl_4-treated mice	Reduced hepatocyte apoptosis, and decreased hepatic inflammation and fibrosis	Manuelpillai et al. Cell Transplant 2010 (13)
Liver fibrosis induced in rats through the bile duct ligation	Amniotic membrane application reduced the severity of fibrosis significantly	Sant'Anna et al. Cell Transplant 2011 (22)
Liver fibrosis induced in rats through the bile duct ligation	Both fresh and cryopreserved human amniotic membrane application decreased the severity of fibrosis significantly	Ricci et al. Cell Tissue Bank 2013 (23)
