# Supplementary figures and images for: Placental Amniotic Epithelial Cells and Their Therapeutic Potential in Liver Diseases
Source: Front Med (Lausanne). 2014 Dec 8;1:48. doi: 10.3389/fmed.2014.00048 (PMC4291892; doi:10.3389/fmed.2014.00048)

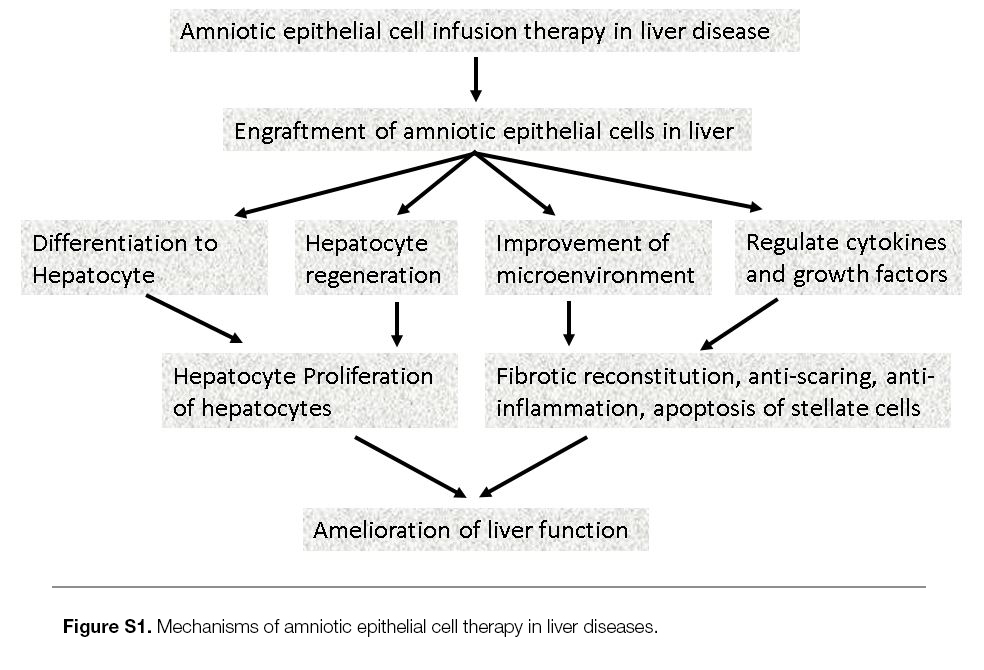

Supplement: Supplementary file 2 [file Image_1.JPEG]
